# Partial Agonists and Dual Disorders: Focus on Dual Schizophrenia

**DOI:** 10.3389/fpsyt.2021.769623

**Published:** 2021-12-16

**Authors:** Lola Peris, Nestor Szerman

**Affiliations:** ^1^Centre Neuchâtelois De Psychiatrie, Marin-Epagnier, Switzerland; ^2^Hospital Universitario Gregorio Marañon, Madrid, Spain; ^3^Fundación Patología Dual, WPA Section on Dual Disorders, WADD, Madrid, Spain

**Keywords:** dual disorders, addiction, partial agonists, schizophrenia, antipsychotics

## Abstract

Dual disorder is a term applied to patients with an addictive disorder and other mental disorder. Epidemiological studies have established that dual disorders are an expectation rather than an exception. They are difficult to diagnose and treat and constitute a huge burden for both patients and their relatives and society. Current treatments are a combination of those needed to treat the addictive disorder with those focused on the co-occurring psychiatric disorder. Focusing specifically on schizophrenia, growing scientific evidence supports the existence of a shared vulnerability for substance use in these patients and those at risk. Various antipsychotics have been found to be useful in the treatment of psychotic symptoms and disorders; however, few effective treatments have been identified until now for substance use disorders in patients with dual schizophrenia. Partial agonism stands as a new pharmacological option available in recent years. Molecules with this kind of action may act as functional agonists or as antagonists, depending on the surrounding levels of the neurotransmitter. Studies have found their efficacy in schizophrenia, addiction, anxiety and depression. Certain partial agonist antipsychotics seem to have a role in the treatment of dual schizophrenia. That could be the case with cariprazine. Because of its higher affinity for dopaminergic D3 receptors compared to D2, a potential to prevent relapse to addiction, added to its antipsychotic efficacy, has been suggested. Here we briefly review current advances and future directions and introduce some personal insights into the role of partial agonists in co-occurring schizophrenia and substance use.

## Introduction

Research estimates that up to 75% of patients with severe mental illness also have a substance use disorder (SUD) (1). When mental disorders occur with addictive disorders, this clinical condition is referred to as a dual disorder (2).

Dual disorders (DD) are a phenomenon associated with increases in emergency department admissions and psychiatric hospitalizations, higher risk of relapse to drug use and increased likelihood of premature deaths, including those resulting from suicide. The individual, social and public health impact of DD is extremely high, and a comprehensive multidisciplinary and scientific response to the needs of those with these disorders is required. Unfortunately, there are many gaps in the global system, which is ill prepared to meet this challenge (2).

In many countries, the lack of attention to DD is partly driven by the structural differentiation and insufficient coordination between programs to treat SUD and those to treat other mental illnesses. Other contributing factors include the limited psychiatric training on how to diagnose and treat DD, and misinformation about the potential role of psychiatric drugs and their mechanisms of action when choosing the most appropriate treatment. Present treatments include a combination of those needed to treat addictive disorders together with those focused on other psychiatric disorders. Finding more specific treatments would undoubtedly help to improve the evolution of DD patients.

The aim of this perspective paper is to highlight the potential role of the dopaminergic partial agonists in patients with dual schizophrenia.

## Advances in Neuroscience and Treatment Challenges

Epidemiological studies have established that DD are an expectation rather than an exception (3). The symptomatic high co-occurrence supports the fact that both conditions are in some way causally linked (4).

Advances in genetics and precision psychiatry suggest that the co-occurrence of substance use and other mental disorders arises, in part, from a shared genetic etiology (5). Large genome-wide association studies are providing the information needed to investigate genetic research on schizophrenia plus SUD and have yielded increasing evidence of a blurred boundary between schizophrenia and substance use disorder. Significant genetic correlations were found with the majority of analyzed substance use, including smoking, alcohol use, schizophrenia, and risk-taking (6–8).

Neuroscience has revealed that addiction and other mental disorders involve a set of interconnected processes that can be targeted strategically, rather than being a disorder primarily defined by a single behavior (as uncontrollable excessive drug use). It is related to interacting neurobiological and environmental factors involved in behaviors of substance and non-substance related disorders (9). Focusing specifically on psychosis and schizophrenia, we should wonder the underlying reasons for the patient's use of substances. As usual, we can find the answers in neuroscience. Growing scientific evidence supports the existence of a shared vulnerability for substance use in these patients and those at risk (10, 11). An increasing number of researchers claim that, if schizophrenia and SUD share genetic underpinnings, this will strongly challenge the rigid diagnostic boundaries that separate these psychiatric disorders, which may have clinical implications. Understanding the pathogenesis of DD as one entity should finally have the potential to improve clinical outcomes and treatments (6).

When treating a patient with dual psychosis, a psychiatrist might wonder whether all antipsychotics are equally effective. From a neuroscience perspective, it currently seems clear that there are many phenotypes of schizophrenia and many antipsychotics with different mechanisms of action. Dual schizophrenia appears to have a different phenotype that requires a new approach.

Most studies indicate that antipsychotics produce a clear improvement in psychotic symptoms, with a more controversial effect on SUD. The possibility that conventional D2 antagonist antipsychotics increase substance craving has been described, suggesting the need for a new approach to improve SUD in patients with schizophrenia. Conversely, treatment retention is generally low, due in part to the intrinsic characteristics of the addiction itself but also to the lack of efficacy and/or potential adverse events.

Interestingly, clozapine has previously been considered the most effective antipsychotic for these dual psychotic patients, and preliminary but consistent data suggest that it limits substance use in them (12). Given the multireceptorial action of this drug, there is no clear explanation for this clinical effect; however, its use is less frequent than expected, which may be related to specific side effects that require monitoring.

Neuroscience based Nomenclature (NbN) is a new system for classifying psychotropic drugs based on their pharmacological profile. The NbN was developed to replace the current indication-based nomenclature and to provide an up-to-date and more useful framework to better inform pharmacological decisions (13). Not all antipsychotics have the same mechanism of action and therefore should have different clinical effects; in the field of SUD, medications are frequently labeled according to their main symptomatic effect (e.g., “anticraving drugs”) or according to imprecise and sometimes old concepts related to treatment strategies (e.g., “replacement therapies, ”“antabuse drugs,” or “substitution treatments”). In contrast, the NbN offers a clearer and more consistent rationale, according to which the main element of classification is based on the pharmacological mechanism of action (14). In addition, pharmacologically driven nomenclature, by highlighting pharmacological domains and mechanisms of action, may increase drug adherence, as it clarifies the rationale for selecting a specific psychotropic agent.

Moreover, from a precision psychiatry perspective, it is important to consider not only the mechanism of action of different drugs but also their effect on brain functional organization, which varies between individuals and changes according to the psychopathological context. From this perspective, psychoactive drugs, including antipsychotics, may have distinct effects depending on individual brain differences (4).

## Partial Agonists in Schizophrenia and SUD

Drugs approved for the treatment of psychiatric disorders often elicit side effects that may limit their use and their acceptance by patients. Partial agonists (PA) used to treat troubles as hypertension, have demonstrated a better profile regarding adverse events. This has fueled research of potential PA for psychiatric treatment, with a good profile of efficacy and limited adverse events. These molecules may act as functional agonists or as antagonists depending on surrounding neurotransmitter levels, and their use appears to result in fewer side effects than full agonists or antagonists without compromising clinical efficacy (15). They stand now as a new pharmacological option and, while their number is still scarce, they have already shown their efficacy in several psychiatric disorders, such as schizophrenia, addiction, anxiety, and depression. They are safe, well-tolerated, and may give rise to a stabilization of the systems. It has been suggested that PA constitute in some way a novel approach to the treatment of mental disturbances.

There are various drugs of established use or interest in the field of DD whose mechanism of action is partial agonism; they include buprenorphine, varenicline, nalmefene, aripiprazole, brexpiprazole, and cariprazine. Although the first three drugs are implicated in the treatment of opioids, tobacco and alcohol addiction and the last three in schizophrenia, from the perspective of neuroscience, their clinical action is projected beyond their current indication. Being focused on dopaminergic partial agonists and dual schizophrenia, it is beyond the scope of this review to cover all these drugs in detail.

The recent development of these antipsychotics with new mechanisms of action are promising prospects for dual schizophrenia treatment. Antipsychotics acting as PA behave as functional antagonists in areas with high levels of dopamine (e.g., mesolimbic pathway) but not in areas where dopamine levels are normal (e.g., nigrostriatal and tuberoinfundibular pathways). They are then expected to reduce positive symptoms without producing movement disorders or prolactin alterations (16). By normalizing and stabilizing dopaminergic tone, PA, unlike full dopamine receptor agonists and antagonists, may have reduced abuse liability or disruptive effects on motivated behavior (17).

The involvement of dopaminergic dysfunction in addiction is well-known. While potent dopamine D2 receptor antagonist antipsychotics have been linked to elevated incidence of SUD (e.g., nicotine addiction in smokers with schizophrenia) (18), PA produce substantially less functional antagonism of D2 receptor-mediated neurotransmission than full antagonists (19). Furthermore, the capacity of PA to increase dopamine activity in the mesolimbic dopaminergic pathway and modulate the dopaminergic system might be beneficial for reducing craving, rewarding effects and relapse. The first published reports suggest that specific PA antipsychotics have a potential role in the treatment of dual schizophrenia, which may be the case with long-acting injectable aripiprazole, that showed efficacy against psychotic symptoms as much as addictive ones in patients with schizophrenia and coexisting SUDs in a first multicenter observational study (20).

Cariprazine, a new PA drug, was introduced recently for the treatment of schizophrenia. It is a dopamine D3-preferring D3/D2 receptor PA, serotonin 5HT1A receptor PA and serotonin 5-HT2B and 5-HT2A antagonist. While other atypical antipsychotics may have significant activity at the D3 receptor (D3R), its high potency as an antagonist/PA at the D3 highlights its unique pharmacological profile among other antipsychotics (21).

Outside its non-psychiatric clinical implications, D3R is known to be involved in schizophrenia, depression, anxiety, and addiction and is found mainly in brain areas regulating cognitive and emotional functions, and reward-related behaviors (22). Preclinical evidence from several animal models of human addiction supports the D3R as a viable target for SUD treatment development and predict that D3R selective antagonists and PA may be effective in addiction treatment by regulating the motivation to self-administer drugs and disrupting drug-associated cue-induced craving (23, 24).

Although the role of D3R in addiction is well-recognized today and it has long been a target in addiction pharmacotherapy, translation to clinical medication development has been challenging until recently, especially in relation to the absence of clinically available D3R preferential compounds. Researchers have discovered highly selective D3R antagonists, PA and full agonists that have worked as crucial tools for pharmacological investigations, including at the behavioral level; however, suggestions have been made to reconsider animal models to achieve translation of preclinical findings to clinical success, or the need to explore additional behavioral models of addiction (25).

One arising issue refers to the optimal timing of administration of treatments; for instance, D3R antagonism may not affect the primary reinforcing effects of the drugs but will reduce the motivation for self-administration. D3R PA may possibly become more effective when drugs are not available, and their behavioral pharmacology appears to be different depending on whether the subjects are drug-naive or have a drug history (25).

## Discussion

There has been extensive research on the psychopharmacological treatment of patients with psychosis and co-occurring SUD, but without significant results until now with the exception of clozapine, although its use is still controversial. This perspective paper describes current trends in the treatment of dual psychosis/schizophrenia with a focus on PA drugs to optimize outcomes and foster the development of new dual schizophrenia treatments. The heterogeneity of the pathophysiology of the various domains of dual schizophrenia requires a diversity of treatments that may currently be best met by the use of PA by expert clinicians. In this respect and beyond some evidence regarding aripiprazole, cariprazine, with a stronger D3R-preferential activity, has shown to be potentially useful in preclinical models of drug use.

Although there has been interest in D3R in addiction treatment for over 20 years, there is a lack of positive results to translate to the clinical field. In recent years, reports and reflections regarding the models used in research in the addiction pharmacotherapy field, as well as the findings about the actions of different types of compounds on the receptors, optimal time of administration and relevance of the patient's consumption trajectory in terms of the efficacy of treatments, will undoubtedly modify this situation. New compounds being tested today or in the near future will likely follow a different pathway to unravel their true potential in the field of addiction and DD treatment.

We can't ignore the fact that the Food and Drug Administration and other health authorities have issued a warning regarding the use of one PA such as aripiprazole and the development of rare impulse control problems, including pathological gambling, binge-eating disorder, and hypersexuality (26). This effect could be a consequence of the increased availability of dopamine (DA) in the brain's reward system. Nevertheless, this is insufficient to explain these effects, and research studies indicate that some clinical phenotypes affected with specific frontal dysfunctions are more vulnerable to develop impulse control disorders when taking dopaminergic agonists (27).

It is conceivable that the benefits of enhancing DA activity to counteract psychopathological symptoms outweigh the risk of such an exceptional side effect in these dual schizophrenia patients. Therefore, the use of PA could be a strength instead of a weakness in dual psychosis, since they may protect against psychotic symptoms and improve addictive ones. It is possible that new PA as cariprazine, with its high antagonist/partial agonist potency at the D3 receptors, minimize these risks while becoming a potential new treatment preventing addiction relapse added to its antipsychotic efficacy, as suggested in previous studies.

Clinical trials, intended to explore the interesting potential of PA in dual schizophrenia and considering recent preclinical findings are warranted.

## Data Availability Statement

The original contributions presented in the study are included in the article/supplementary material, further inquiries can be directed to the corresponding author.

## Author Contributions

LP and NS contributed equally to the conception, drafting and critical revision of the work, and provided approved for the publication of the content. All authors contributed to the article and approved the submitted version.

## Conflict of Interest

LP has received honoraria for being a speaker for Recordati and Janssen. NS has received honoraria for being a speaker for Janssen, Rovi, Lundbeck, Otsuka and Takeda, and consultant/member of advisory boards for Lundbeck and Takeda.

## Publisher's Note

All claims expressed in this article are solely those of the authors and do not necessarily represent those of their affiliated organizations, or those of the publisher, the editors and the reviewers. Any product that may be evaluated in this article, or claim that may be made by its manufacturer, is not guaranteed or endorsed by the publisher.
